# The Role of Adrenergic and Angiotensinergic Systems in Vascular Effect of Alcoholic of Extract *Trigonella foenum-graecum* Seed in Diabetic Rats

**Published:** 2011

**Authors:** Mohammad Reza Vaez Mahdavi, Mehrdad Roghani, Tourandokht Baluchnejadmojarad

**Affiliations:** a *Department of Physiology, School of Medicine and Medicinal Plant Research Center, Shahed University, Tehran, Iran.*; b *Department of Physiology, School of Medicine, Tehran University of Medical Sciences, Tehran, Iran.*

**Keywords:** Trigonella foenum-graecum, Adrenergic, Angiotensinergic, Diabetes mellitus, Aorta

## Abstract

Considering the anti-diabetic potential of *Trigonella foenum-graecum *(TFG) and its beneficial effect on aortic reactivity of diabetic rats, this study was conducted to evaluate the effect of its alcoholic seed extract on aortic reactivity and also figure out mechanisms including the role of adrenergic and angiotensinergic systems in streptozotocin-diabetic rats. Male Wistar rats were divided into control, extract-treated control, diabetic, and extract-treated diabetic groups. Diabetes was induced by a single IP injection of streptozotocin (STZ; 60 mg/kg). Treatment groups received TFG extract (200 mg/kg; IP) every other day for 1 month. Contractile response of thoracic aorta to KCl and noradrenaline (NA) was then determined. For determination of the involvement of adrenergic and angiotensinergic systems, rings were incubated before the experiments with prazocin, propranolol, and/or captopril and then NA- and angiotensin I (Ag-I)-induced contractions were recorded. Diabetic state significantly increased maximum contractile response to KCl and NA (p < 0.01-0.005) comparing to control groups and treatment with TFG extract in diabetic group significantly improved these changes comparing to untreated diabetic group (p < 0.05-p < 0.01). On the other hand, pretreatment of prazosin and propranolol caused a significant reduction in contractile response of all groups (p < 0.05-0.001) meanwhile was no significant difference among the groups. In addition, pre-incubation with captopril caused a significant reduction in contractile response of TFG-treated diabetic group comparing untreated diabetic group (p < 0.05). Finally we concluded that alcoholic seed extract of TFG could improve some functional indices of the vascular system in diabetic state and angiotensinergic system is partly involved in this response.

## Introduction

Mortality from cardiovascular abnormalities including hypertension, atherosclerosis, microangiopathy, and congestive heart failure is almost three times more prevalent in the diabetic than the general population (1, 2). Therefore, finding new treatment strategies for attenuation of diabetic vascular complications have been always a mainstay in medicine. In this regard, *Trigonella foenum-graecum *(TFG; fenugreek) was considered as an appropriate candidate. *TFG *is a plant with traditional medicinal use in diabetes. Beneficial effects have so far been demonstrated in diabetic animals and in both insulin and non-insulin-dependent diabetic subjects (3). Hypoglycemic and anti-hyperglycemic effects of fenugreek seed (4) and its aqueous leaf extract (5) have previously been reported in experimentally induced diabetes rats. In addition, endothelium-dependent attenuating effect of this plant in aortic rings from diabetic rats has already been reported (6). Besides, endothelium-dependent and relaxant effect from alcoholic extract of fenugreek were also proven (7). Therefore, the present study was carried out to evaluate the role of adrenergic and angiotensinergic systems for their beneficial effects on vascular reactivity of thoracic aorta of STZ-diabetic rats.

## Experimental

*Preparation of TFG extract*

Fenugreek seed was obtained from local grocery (Tehran) in June, and was systemically identified at the Department of Botany of Shaheed Beheshti University (Voucher number: 2005-46). Then, seeds were washed, dried under shade at room temperature, and finely powdered with a grinder. Thereafter, 100 g of powder was suspended in 1: l of methanol for 48 h in dark (percolation method). The extract was then filtered and concentrated to obtain the solid residue which was and refrigerated until further use. The yield of this process was 19.5% (w/w). Fenugreek extract of lower concentrations of Fenugreek extract were prepared by dilution of the stock with cold and sterile 0.9% saline solution.

*Animal experiments*

Male albino Wistar rats (Pasteur’s institute, Tehran, Iran) weighing 245-285 g (10-12 weeks old) were housed in an air-conditioned colony room at 23 ± 1 °C and supplied with standard pellet diet and tap water ad libitum. Procedures involving animals and their care were conducted in conformity with NIH guidelines for the care and use of laboratory animals.

The animals (n = 30) were randomly divided into four experimental groups: vehicle-treated control (VC, n = 7), extract-treated control (EC, n = 7), vehicle-treated diabetic (VD, n=7), and extract-treated diabetic (ED, n = 9). Diabetes was induced by a single intraperitoneal injection of streptozotocin (STZ, 60 mg/Kg dissolved in cold 0.9% saline) immediately before test. Control and extract-treated control animals received normal saline solution and alcoholic extract of fenugreek extract (200 mg/Kg, IP) respectively. This dose was chosen on the basis of our pilot dose-response studies. The extract was administered one other day to extract-treated diabetic animals from day 3 after diabetes induction. Serum glucose level and body weight were measured one week before and also four weeks after the experiment. Diabetes was verified by a non-fasting serum glucose level higher than 250 mg/dL using glucose oxidation method (glucose oxidase kit, Zistchimie, Tehran, Iran). All of the abovementioned were treatments continued for one month. 

*Experimental procedure*

The routine protocol was applied as described before (8-9). Briefly, after being anesthetized, descending thoracic aorta of the animal was carefully excised and placed in a petri dish filled with cold Krebs solution containing (in mM): NaCl 118.5, KCl 4.74, CaCl_2_ 2.5, MgSO_4_ 1.18, KH_2_PO_4_ 1.18, NaHCO_3_ 24.9, and glucose 10.0 (8). The aorta was then cleaned of excess connective tissue and fat and cut into rings of approximately 4 mm in length. One ring of each pair was left intact, and in the other ring, endothelium was mechanically removed by gently rotating it on a glass micropipette. Aortic rings were suspended between the bases of two triangular-shaped wires. One wire was attached to a fixed tissue support in a 50 mL isolated tissue bath containing Krebs solution (pH 7.4) maintained at 37°C and continuously aerated with a mixture of 5% CO_2_ and 5% O_2_. The other end of each wire was attached by a cotton thread to a F60 isometric force transducer, which was connected to A/D board of IBM-compatible computer. Recording and analysis of data was performed using the software Physiograph I (Behineh Arman Co., Tehran, Iran). The rings were allowed to equilibrate for 60 min under a resting tension of 2 g before experiments were begun. During equilibration period, the rings were washed every 30 min. Successful removal of the endothelium was confirmed by loss of acetylcholine (10^-5^ M)-induced relaxation in preconstricted rings by NA (10^-6^ M). Concentration-response curves were obtained with KCl and thereafter with NA in aortic rings with or without endothelium. In this regard, KCl (10-50 mM) and NA (10^-9^-10^-4^ M) were added in a cumulative manner until a maximum response was achieved. 

To determine the involvement of adrenergic and angiotensinergic systems, rings were incubated 1 min before the experiments with *α*1-adrenoceptor blocker, prazosin (10 nM) and *β*-adrenoceptor blocker propranolol (10 nM) and then contractile response to NA at a submaximal concentration (1 μM) were carried out. Meanwhile, some rings were incubated 5 min before the experiment with angiotensin-converting enzyme inhibitor captopril (10 μM) and then angiotensin I (100 nM)-induced contraction was recorded. 

After each experiment, aortic rings were dried at 45°C for 5 min, weighed, and cross-sectional area (CSA) were calculated using the following formula: Cross-sectional area (mm^2^) = weight (mg) × [length (mm) × density (mg/mm^3^)]^-1^. The density of the preparations was assumed to be 1.05 mg/mm^3^ (10).

*Drugs and chemicals*

Noradrenaline, acetylcholine-HCl, prazosin, and SNP were purchased from Sigma Chemical (St. Louis, Mo., USA). Streptozotocin was obtained from Pharmacia and Upjohn (USA). All other chemicals were purchased from Merck (Germany) and Temad (Tehran, Iran). STZ was freshly dissolved in 0.9% saline solution. Prazosin was dissolved in DMSO. 

*Data and statistical analysis*

All values were given as means ± SEM. Contractile responses to NA, KCl, and Ag I were expressed as grams of tension per cross-sectional area of tissue. Statistical analysis was carried out using student’s paired t-test and one-way analysis of variance (ANOVA) followed by Tukey post-hoc test. A statistical p-value less than 0.05 was considered significant.

## Results

Body weight and serum glucose level were measured before and 4 weeks after the experiment (Table 1). After one month, the body weight of the diabetic rats were found to be significantly decreased comparing to one week before the start of experiment (p < 0.05). Diabetic rats also showed significant elevated serum glucose level (p < 0.001). Treatment of diabetic rats with alcoholic extract of fenugreek (200 mg/kg) caused a significant lower glucose serum level (p < 0.01) and a non-significant higher body weight in extract-treated diabetic rats as compared to diabetic group. Furthermore, there was a significant reduction in cross-sectional area of aortic rings in diabetic group (p < 0.05) and extract-treated diabetic group did not show any significant improvement (data not shown).

**Table 1:** Body weight and serum glucose level of control, diabetic, and TFG extract-treated diabetic rats.

	**Body weight (g)**		**Serum glucose (mg/dl)**	
	**Before**	**After**	**Before**	**After**
Control*	268.3 ± 4.9*	279.6 ± 5.5	138.7 ± 4.8**	141.3 ± 4.6*
Control + TFG	271.6 ± 4.8*	254.3 ± 5.1	136.2 ± 5.4*	117.2 ± 5.9*
Diabetic*	274.3 ± 6.4*	219.8 ± 4.9**	142.3 ± 4.9*	389.1 ± 13.7***
Diabetic + TFG	256.5 ± 5.2*	237.2 ± 5.2	151.7 ± 4.1	243.2 ± 10.6**#

* p < 0.05, ** p < 0.005, *** p < 0.001 (In comparison with control group) # p < 0.01 (In comparison with diabetic group

**Figure 1:**
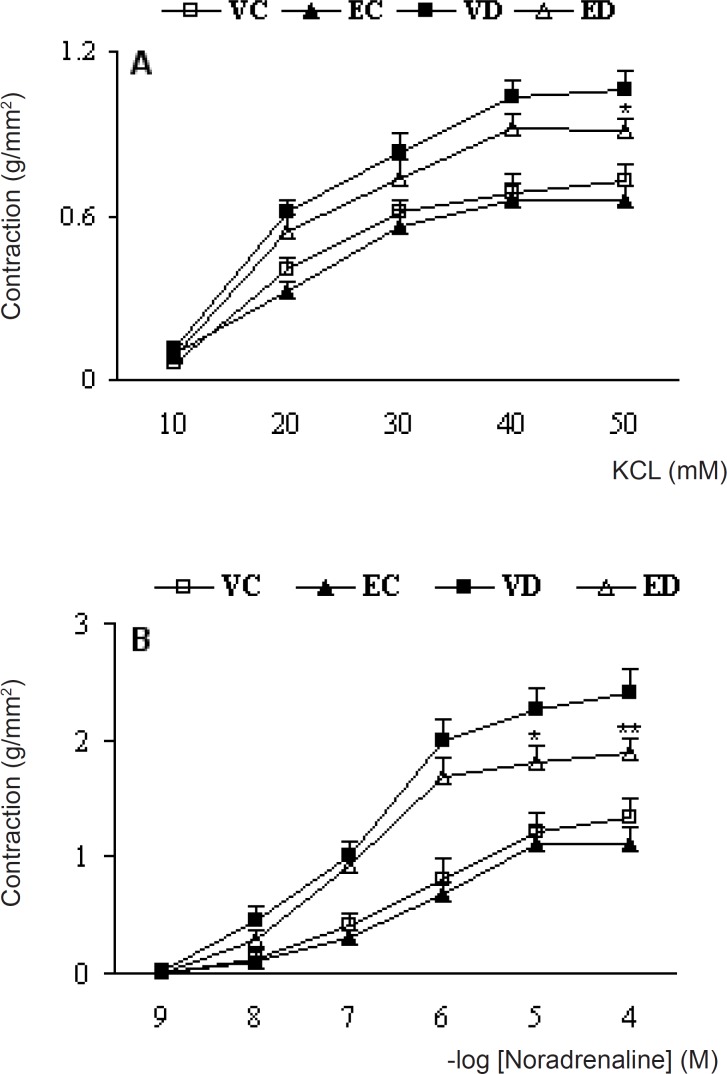
Cumulative concentration-response curves for KCL (A) and NA (B) in endothelium-intact aortic preparations 1 month after the exprement . Contractile responses are expressed as grams of tension per cross sectional area (mm^2^). Data are shown as means ±SEM. ^*^P<0.05, ^**^P<0.01(Compared to VD) (VC, EC, VD and ED represent vehicle-treated control . extract- treated control , vehicle-treated diabetic, and extract- treated control, dose of 200 mg/kg of the extract)

Cumulative addition of KCl (10-50 mM) and NA (10^-9^-10^-4^ M) to the organ bath resulted in concentration-dependent contractions in aortas of all groups (Figure 1). 

The contractile responses to KCl at concentrations higher than 20 mM in diabetic rats were found to be significantly higher than control rats and treatment of diabetic rats with TFG extract caused a significant reduction in contractile response to KCl at a concentration of 50 mM. The calculated, EC50 for this response was 18.1 and 16.4 mM for endothelium-intact untreated diabetic and treated-diabetic rats and there was no significant difference between the groups in this respect. The contractile responses to NA at concentrations higher than 10^-7^ M in vehicle-treated diabetic rats were found to be significantly higher than vehicle-treated control rats and treatment of diabetic rats with TFG extract caused a significant reduction in contractile response to NA. Meanwhile, EC50 for this response was 0.82 and 0.86 M for endothelium-intact untreated diabetic and treated-diabetic rats and there was also no significant difference between the groups in this regard. Furthermore, treatment of control rats with TFG extract did not produce any significant changes in response to KCl and NA. 

**Figure 2:**
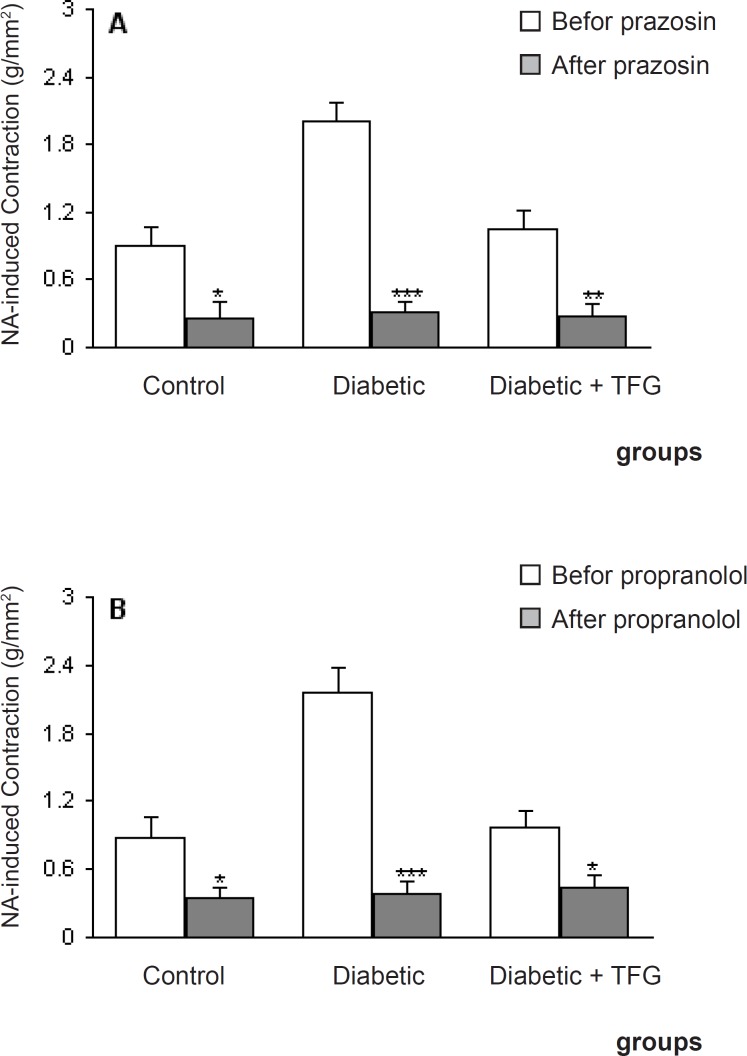
Maximum NA-induced contractile response of endothelium-intact aortic rings different groups 1 month after the experiment in the absence and presence of prazosin propranolol (B). Data are shown as means ± SEM. ^*^p < 0.05, ^**^ p < 0.01, ^***^ p < 0.001 (as compared to data before addition of prazosin propranolol (10 nM)).

**Figure 3:**
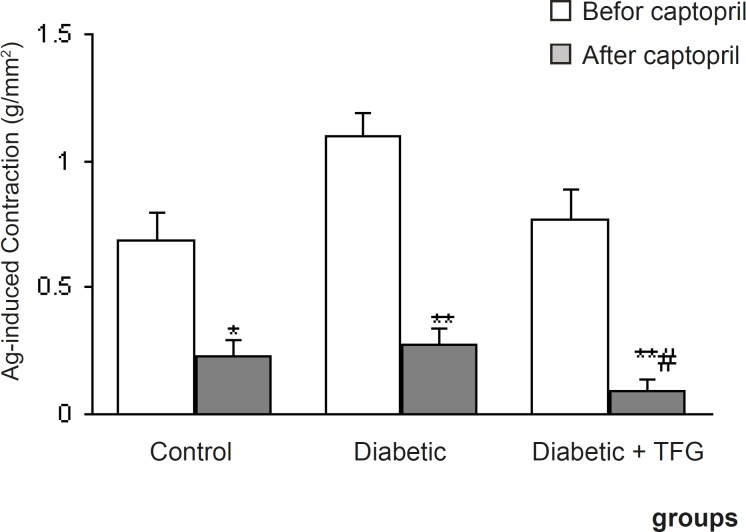
Maximum angiotensin I-induced contractile response of endothelium-intact aortic rings from different groups 1 month after the experiment in the absence and presence of captopril. Data are shown as means ± SEM. ^*^p < 0.01, ^** ^p < 0.005 (relative to data before addition of the captopril); # p < 0.05 (as compared to diabetic group

Pretreatment of aortic rings with prazosin and propranolol caused a significant reduction in contractile response of all groups (p < 0.05-0.001) (Figure 2) and there was no significant difference among the groups in this regard. On the other hand, pre-incubation with captopril caused a significant reduction in contractile response of TFG-treated diabetic group comparing to untreated diabetic group (p < 0.05) (Figure 3). In this respect, there was also no significant difference regarding EC50. 

## Discussion

The results of the present study demonstrated that aortas from 1-month STZ-diabetic rats are more responsive to the contractile effect of adrenoceptor agonist NA and to non-specific agent KCl than those from corresponding controls. Similar results showing the increased vascular responsiveness to contractile agents in STZ-diabetic rats were reported in most previous studies (10, 11). This increased vascular smooth muscle responsiveness in diabetic rats could be attributed to deficient endothelial activity (10, 11), enhanced phosphoinositide (PI) metabolism (12), enhanced sensitivity of calcium channels (8), and increased sensitivity to adrenergic agonists (13). Furthermore, oxidative stress is increased due to excessive production of oxygen-free radicals and decreased antioxidant defense systems (14-16). Although initially we expected no change in contractile response following pretreatment of aortic rings with propranolol as a classic beta receptor antagonist, but its addition caused a reduction in contractile response to NA. This may partly be attributed to its calcium-channel blocking activity and not its beta receptor blocking activity (17). 

In this study, alcoholic seed extract of fenugreek attenuated the increased responsiveness of aortic rings in diabetic state. Our results demonstrated that fenugreek extract at a dose of 200 mg/kg could partially counteract the increased contractile response of endothelium-intact aortic rings of diabetic rats following NA and/or KCl administration. The beneficial effect of chronic fenugreek extract treatment on NA- and KCl-induced contractions was specific for aortas of diabetic rats, because the extract treatment did not produce any significant change in control preparations. The results of this study also showed that the beneficial effect of TFG extract in aorta of diabetic rats is partly mediated through pathway of angiotensinergic system and there is no significant change in the adrenoceptor system activity. 

To conclude, the findings obtained in this study indicate that chronic treatment of diabetic rats with alcoholic seed extract of fenugreek could partially prevent the development of changes in vascular reactivity and angiotensinergic system which in part are essential in this matter.
